# The effect of corneal power on the accuracy of 14 IOL power formulas

**DOI:** 10.1186/s12886-024-03395-9

**Published:** 2024-03-19

**Authors:** Jialin Xu, Lu Zhang, Er Mo, Kaiyi Zhu, Yitong Zhu, Ke Feng, Zunting Wu, Yangran Zheng, Fang Huang, Xianhui Gong, Jin Li

**Affiliations:** 1 National Clinical Research Center for Ocular Diseases, Eye Hospital, Wenzhou Medical University, Wenzhou, 325027 China; 2 Eye Hospital, School of Ophthalmology and Optometry, Wenzhou Medical University, Wenzhou, Zhejiang China; 3 Eye Hospital of Wenzhou Medical University Hangzhou Branch, 618 East Fengqi Road, Hangzhou, Zhejiang 310000 China; 4 Wenzhou Medical University Eye Hospital, 270 West Xueyuan Road, Wenzhou, Zhejiang 325027 China

**Keywords:** Cornea power, IOL power calculation, New IOL formula

## Abstract

**Background:**

This study evaluates the impact of corneal power on the accuracy of 14 newer intraocular lens (IOL) calculation formulas in cataract surgery. The aim is to assess how these formulas perform across different corneal curvature ranges, thereby guiding more precise IOL selection.

**Methods:**

In this retrospective case series, 336 eyes from 336 patients who underwent cataract surgery were studied. The cohort was divided into three groups according to preoperative corneal power. Key metrics analyzed included mean prediction error (PE), standard deviation of PE (SD), mean absolute prediction error (MAE), median absolute error (MedAE), and the percentage of eyes with PE within ± 0.25 D, 0.50 D, ± 0.75 D, ± 1.00 D and ± 2.00 D.

**Results:**

In the flat K group (Km < 43 D), VRF-G, Emmetropia Verifying Optical Version 2.0 (EVO2.0), Kane, and Hoffer QST demonstrated lower SDs (± 0.373D, ± 0.379D, ± 0.380D, ± 0.418D, respectively) compared to the VRF formula (all *P* < 0.05). EVO2.0 and K6 showed significantly different SDs compared to Barrett Universal II (BUII) (all *P* < 0.02). In the medium K group (43 D ≤ Km < 46 D), VRF-G, BUII, Karmona, K6, EVO2.0, Kane, and Pearl-DGS recorded lower MAEs (0.307D to 0.320D) than Olsen (OLCR) and Castrop (all *P* < 0.03), with RBF3.0 having the second lowest MAE (0.309D), significantly lower than VRF and Olsen (OLCR) (all *P* < 0.05). In the steep K group (Km ≥ 46D), RBF3.0, K6, and Kane achieved significantly lower MAEs (0.279D, 0.290D, 0.291D, respectively) than Castrop (all *P* < 0.001).

**Conclusions:**

The study highlights the varying accuracy of newer IOL formulas based on corneal power. VRF-G, EVO2.0, Kane, K6, and Hoffer QST are highly accurate for flat corneas, while VRF-G, RBF3.0, BUII, Karmona, K6, EVO2.0, Kane, and Pearl-DGS are recommended for medium K corneas. In steep corneas, RBF3.0, K6, and Kane show superior performance.

## Introduction

In cataract surgery, achieving precise refractive outcomes is crucial and largely depends on accurate ocular biometric measurements. One of the key factors in this process is the selection of appropriate intraocular lens (IOL) power calculation formulas. Despite advancements in technology and methodology, prediction errors (PE) continue to pose challenges, particularly in cases with unique ocular characteristics like axial length (AL), anterior chamber depth (ACD, measured from corneal epithelium to lens), corneal power, and lens thickness (LT) [1]. 

The development of newer IOL power calculation formulas such as the Barrett Universal II(hereafter BUII) [2], Castrop [3], Emmetropia Verifying Optical Version 2.0(EVO2.0), Hoffer QST [4], Kane [5], Karmona [6], Cook K6(K6), Naeser2 [7], Olsen C [8, 9], Pearl­DGS [10], Radial Basis Function Version 3.0 (RBF3.0), T2 [11], VRF [12] and VRF-G [13] marks significant progress in this field [13–16]. These formulas have shown enhanced accuracy, especially in eyes with atypical AL, [17–19]ACD, [19] and other specific ocular parameters [20]. 

While AL and ACD are often highlighted in IOL power calculation, the role of corneal power in influencing the accuracy of these formulas is equally critical. Studies with comprehensive datasets have highlighted that variations in corneal power can significantly affect the precision of IOL power calculation formulas [21–25]. Traditional comparisons primarily focused on the performance of third- and fourth-generation formulas in eyes with atypical corneal power, revealing limitations in formulas like Haigis [26], Hoffer Q [27], and SRK/T [28] in cases of steep corneal power [22, 24, 25]. However, evaluations of the newer-generation formulas, such as RBF and Olsen C, suggest substantial improvements in accuracy [21, 22]. 

This study aims to examine the influence of corneal power on the prediction accuracy of a range of IOL calculation formulas. By comparing newer IOL power calculation formulas (BUII, Castrop, EVO2.0, Hoffer QST, Kane, Karmona, K6, Naeser2, Olsen (OLCR), Pearl-DGS, RBF3.0, T2, VRF, and VRF-G), we intend to provide insights for selecting the most suitable IOL formula based on individual corneal characteristics. This approach will enhance our understanding of the relationship between corneal power and IOL formula accuracy, aiding in more precise postoperative refractive outcomes.

## Materials and methods

### Patients and measurements

This retrospective case series study was conducted from January 2019 to December 2021 at the Eye Hospital of Wenzhou Medical University. The patient cohort consisted of individuals who underwent uncomplicated cataract surgery via phacoemulsification. The surgeries were performed by two experienced cataract surgeons, LJ and HF, with each patient receiving the same intraocular lens (IOL) model (SN6CWS, Alcon, Fort Worth, TX, USA). Selection criteria for study participants were aligned with the IOL power calculation guidelines proposed by Hoffer et al. in 2020 [29]. In cases where patients underwent sequential bilateral cataract surgery, the right eye was preferentially included in the study. Inclusion criteria were a postoperative corrected distance visual acuity of at least 20/40. Exclusion criteria encompassed patients with a history of eye disease, prior ocular surgery, invalid biometry, intraoperative or postoperative complications, or lack of postoperative manifest refraction data.

Preoperative ocular parameters were measured using the Lenstar LS900 (Haag-Streit AG, Koeniz, Switzerland Biometry: v2.5.2, IOL: v4.2.1), covering axial length (AL), anterior chamber depth (ACD), flat and steep keratometry readings (K1 and K2), central corneal thickness (CCT), horizontal corneal diameter (CD), and lens thickness (LT). The average of the keratometry reading (Km) was calculated from the flat and steep keratometry readings. Postoperative manifest refraction with a constant distance of 6 m from the phoropter to the optotype screen was assessed between one and three months after surgery to ensure stabilization of refractive outcomes.

### IOL power calculation

The study utilized a range of formulas for spherical equivalent prediction, including BUII, Castrop, EVO2.0, Hoffer QST, Kane, Karmona, K6, Naeser2, Olsen (OLCR), Pearl-DGS, RBF3.0, T2, VRF, and VRF-G. Several of these formulas (Hoffer QST, Kane, RBF3.0, VRF-G) also incorporated gender in their calculations [30]. 

Refractive prediction error (PE) was determined by comparing the spherical equivalent of the postoperative manifest refraction with each formula’s predicted spherical equivalent using the IOL power actually implanted. The standard deviation (SD) of PE’s error was calculated, with positive and negative PE values indicating hyperopic and myopic shifts, respectively. The mean refractive prediction error (ME), mean absolute error (MAE), median absolute error (MedAE), and the percentages of eyes with PE within ± 0.25 D, ± 0.5 D, ± 0.75 D, ± 1.00 D and ± 2.00 D were also computed.All formulas were individually optimized by their respective authors to achieve a mean PE of zero.

### Statistical analysis

The data were analyzed with the SPSS software (version 25.0, IBM Corp.) and R Project for Statistical Computing (https://www.r-project.org). The Kolmogorov-Smirnov test assessed data normality. The PE of Castrop, Karmona, and Olsen (OLCR) showed normal distribution, while nonparametric Wilcoxon tests were applied to the PE of all formulas. Heteroscedastic method [31]was used to evaluate SD, MAE, MedAE, and the proportion of eyes within different diopter ranges. The Holm-Bonferroni correction was applied for multiple comparisons to determine adjusted P-values. A P-value of less than 0.05 was considered statistically significant.

## Results

This study included 336 eyes from 336 patients, with an average participant age of 70.29 years (range 33–87 years), predominantly women (66.1%, *n* = 222) and right eyes (64.3%, *n* = 216). Pre-surgical biometric ocular parameters are detailed in Table 1. Based on mean keratometry (Km), patients were categorized into three groups: flat K (Km < 43 D), medium K (43 D ≤ Km < 46 D), and steep K (Km ≥ 46 D). Both the flat and steep K groups represented around 20% of the cohort each.

**Table 1:** Preoperative patient biometric ocular parameters

Parameter	Mean ± SD/ Median (IQR)	Range
Axial length, mm*	23.33 (0.7)	21.21–31.98
Anterior chamber depth, mm	3.01 ± 0.44	1.69–4.28
Flat keratometry (K1), D	44.14 ± 1.71	39.77–49.36
Steep keratometry (K2), D	44.85 ± 1.71	40.65–50.76
Mean of keratometry (Km), D	44.49 ± 1.68	40.21–50.06
Corneal central thickness, µm	535.09 ± 34.13	439–649
Horizontal corneal diameter, mm	11.54 ± 0.46	9.89–13.07
Lens thickness, mm	4.47 ± 0.49	2.77–5.88
Axial length distribution, n (%)		
AL<22.0 mm	29	8.63%
22.0 mm ≤ AL<26.0 mm	289	86.01%
AL ≥ 26.0 mm	18	5.36%
Keratometry subgroups, n (%)		
Km<43.0D (Flat)	65	19.35%
43.0D ≤ Km<46.0D (Medium)	205	61.01%
Km ≥ 46.0D (Steep)	66	19.64%

ACD, as measured from the corneal epithelium to the lens

*Data with a non-normal distribution was shown as the median and interquartile range (IQR)

SD, standard deviation; D, diopter; IOL, intraocular lens

### Formula accuracy in all patients

Table 2 summarizes the outcomes for 14 IOL formulas. It details the optimized constants, PE, SD, MAE, MedAE, and the percentage of eyes within specific PE ranges. The ME for all formulas was not significantly different from zero (*P* > 0.05), indicating overall accurate predictions. Formulas with the highest accuracy included K6 (SD ± 0.399D), EVO2.0 (SD ± 0.403D), VRF-G (SD ± 0.403D), Kane (SD ± 0.404D), and RBF3.0 (SD ± 0.404D). The Olsen (OLCR) formula showed the largest SD (± 0.459D), yet no statistical difference was observed in the SDs across all formulas (*P* > 0.05). EVO2.0, Pearl-DGS, RBF3.0, and BUII outperformed in achieving a PE within ± 0.25D, with over 53% of eyes falling in this category. In contrast, Olsen (OLCR), Castrop, VRF, and T2 had less than 50% of eyes reaching a PE within ± 0.25D.

**Table 2:** Refractive outcomes and optimized constants obtained by each formula in all eyes

Formula	Optimized Constants	PE	SD	MAE	MedAE	Eyes within PE (%)				
						PE ≤ 0.25 D	PE ≤ 0.50 D	PE ≤ 0.75 D	PE ≤ 1.00 D	PE ≤ 2.00 D
Barrett Universal II	1.940	0.000	0.425	0.319	0.237	53.27	78.87	91.37	97.92	100.00
Castrop	0.420 0.150	0.003	0.442	0.348	0.281	45.24	75.30	89.58	97.02	100.00
EVO 2.0	119.068	0.000	0.403	0.306	0.236	54.17	78.87	94.64	98.21	100.00
Hoffer QST	5.620	0.000	0.416	0.318	0.255	50.30	79.46	92.56	97.62	100.00
Cooke K6	119.250	0.000	0.399	0.305	0.234	52.38	80.06	94.35	97.92	100.00
Kane	119.043	0.000	0.404	0.307	0.238	52.38	78.87	93.15	97.62	100.00
Karmona	119.430	0.005	0.415	0.325	0.250	51.79	78.57	93.45	97.92	100.00
Naeser 2	1.439 0.940	0.004	0.447	0.342	0.259	50.30	76.79	90.77	96.43	100.00
Olsen (OLCR)	4.94	0.000	0.459	0.364	0.302	43.75	74.11	89.88	97.02	100.00
Pearl-DGS	119.299	0.000	0.408	0.313	0.243	53.57	77.98	93.15	98.21	100.00
RBF 3.0	119.031	0.000	0.404	0.306	0.233	53.27	80.95	93.15	98.51	100.00
T2	119.034	0.000	0.424	0.327	0.266	49.70	77.38	92.26	96.73	100.00
VRF	5.598	0.001	0.445	0.342	0.273	48.21	75.00	90.77	97.62	100.00
VRF-G	119.088	0.002	0.403	0.305	0.238	52.38	81.55	94.64	97.62	100.00

PE = mean prediction error. SD = standard deviation of the error. MedAE = median absolute error. MAE = mean absolute error. D = diopter

### Formula accuracy according to corneal power

**Fig. 1:**
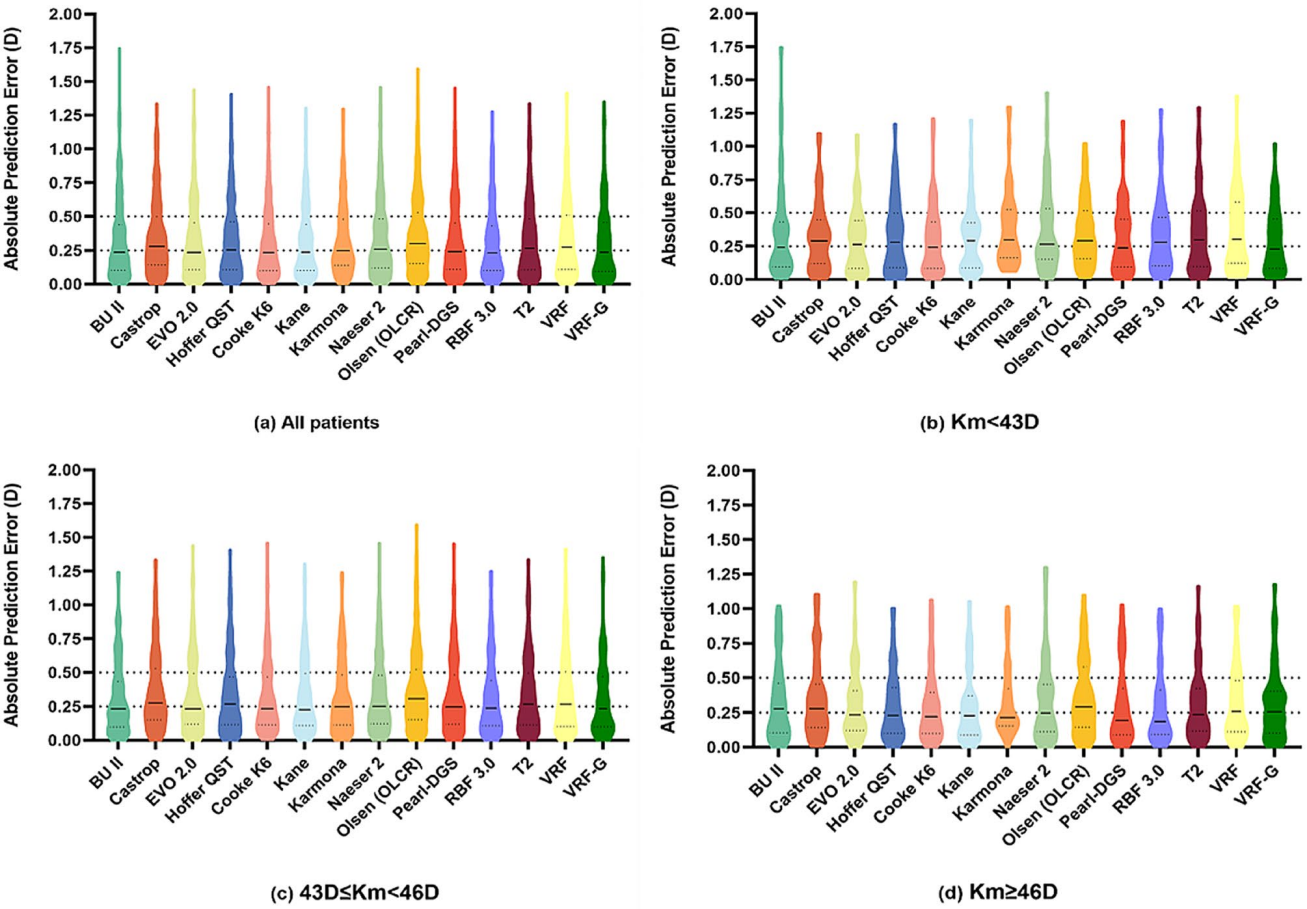
Violin diagrams of absolute prediction error for 14 formulas in all patients and subgroups

**Fig. 2:**
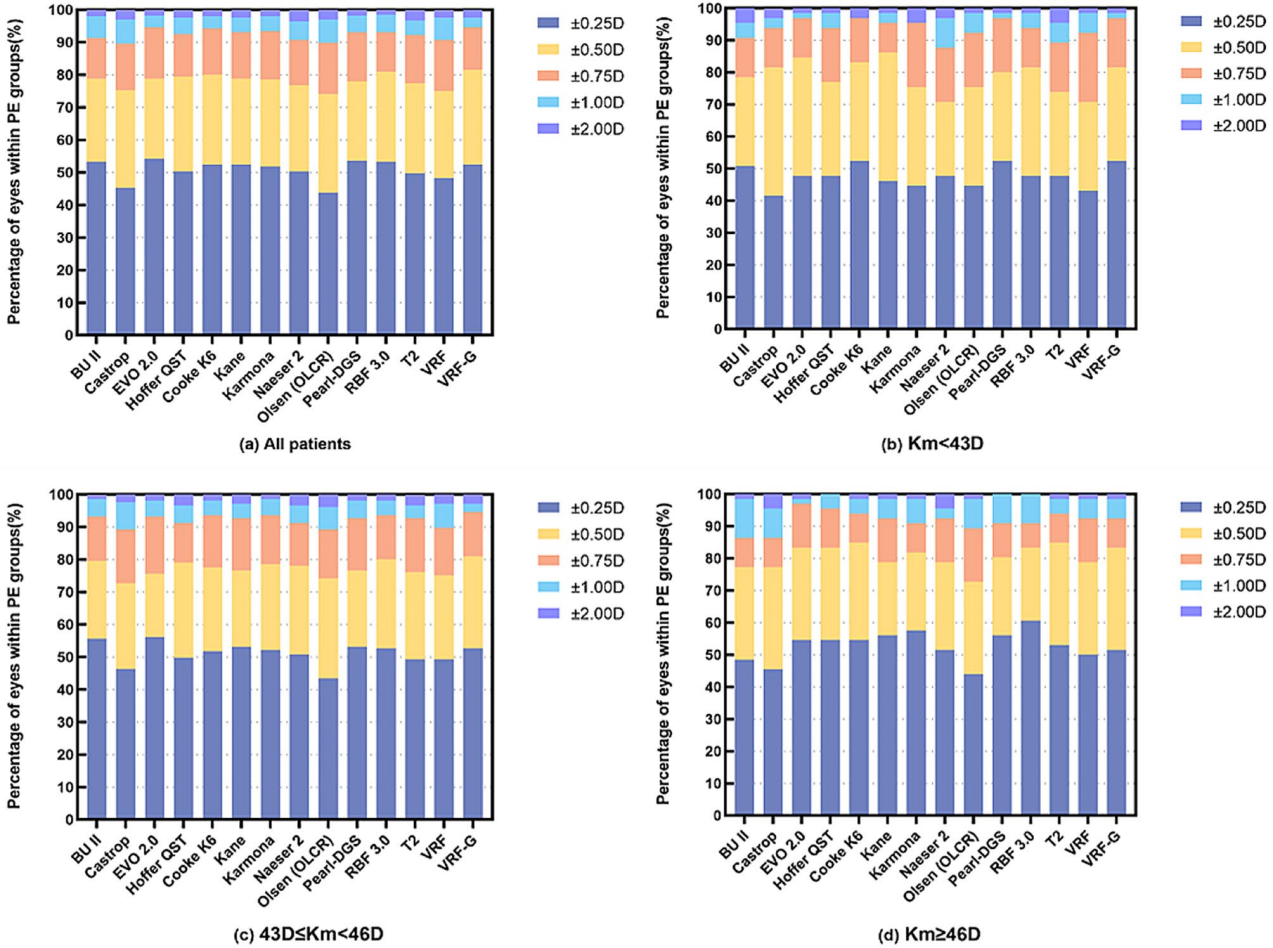
Stacked histogram of percentages with PE within different range in all patients and subgroups

Table 3; Figs. 1 and 2 present the performance of each IOL formula across different corneal power subgroups.

**Table 3:** Predictive outcomes of IOL calculation formulas according to corneal power

	BU II	Castrop	EVO 2.0	Hoffer QST	Cook K6	Kane	Karmona	Naeser 2	Olsen (OLCR)	Pearl-DGS	RBF 3.0	T2	VRF	VRF-G
Km<43.0D(*n* = 65)														
PE	0.037	-0.048	0.014	-0.030	-0.031	-0.054	-0.137	-0.138	-0.139	-0.061	-0.037	0.020	-0.018	-0.047
SD	0.469	0.400	0.379 ^a,b^	0.418 ^a^	0.383 ^b^	0.380 ^a^	0.436	0.453	0.407	0.382	0.418	0.443	0.468	0.373^a^
MAE	0.340	0.318	0.291	0.324	0.293	0.299	0.369	0.368	0.355	0.301	0.326	0.341	0.365	0.294
MedAE	0.243	0.291	0.265	0.280	0.242	0.293	0.299	0.266	0.292	0.239	0.281	0.299	0.303	0.229
Percentage of Eyes within Diopter Range Indicated														
± 0.25D	50.77	41.54	47.69	47.69	52.31	46.15	44.62	47.69	44.62	52.31	47.69	47.69	43.08	52.31
±0.50D	78.46	81.54	84.62	76.92	83.08	86.15	75.38	70.77	75.38	80.00	81.54	73.85	70.77	81.54
± 0.75D	90.77	93.85	96.92	93.85	96.92	95.38	95.38	87.69	92.31	96.92	93.85	89.23	92.31	96.92
± 1.00D	95.38	96.92	98.46	98.46	96.92	98.46	95.38	96.92	98.46	98.46	98.46	95.38	98.46	98.46
43.0D ≤ Km<46D(*n* = 205)														
PE	-0.010	0.005	0.007	0.016	0.007	0.010	0.016	0.003	0.007	-0.006	-0.003	0.008	0.015	0.004
SD	0.409 ^a,c^	0.450	0.417 ^c^	0.426	0.408 ^c,e^	0.415 ^c^	0.406	0.441	0.470	0.414 ^c,e^	0.405 ^a, c,d^	0.429	0.448	0.413
MAE	0.311 ^c,e^	0.356	0.315 ^c,e^	0.324	0.314 ^c,e^	0.315 ^c,e^	0.313 ^c,e^	0.338	0.368	0.320 ^c,e^	0.309 ^a,c^	0.331	0.342	0.307 ^c,e^
MedAE	0.233	0.276	0.233^c^	0.269	0.234	0.227	0.249	0.252	0.308	0.248	0.239	0.267	0.267	0.235
Percentage of Eyes within Diopter Range Indicated														
± 0.25D	55.61	46.34	56.10	49.76	51.71	53.17	52.20	50.73	43.41	53.17	52.68	49.27	49.27	52.68
±0.50D	79.51	72.68	75.61	79.02	77.56	76.59	78.54	78.05	74.15	76.59	80.00	76.10	75.12	80.98
± 0.75D	93.17	89.27	93.17	91.22	93.66	92.68	93.66	91.22	89.27	92.68	93.66	92.68	89.76	94.63
± 1.00D	98.54	97.56	98.05	96.59	98.05	97.07	98.54	96.59	96.10	98.05	98.05	96.59	97.07	97.07
Km ≥ 46D(*n* = 66)														
PE	-0.008	0.049	-0.036	-0.021	0.013	0.022	0.109	0.145	0.116	0.078	0.048	-0.044	-0.025	0.043
SD	0.428	0.448	0.375	0.377	0.380	0.384	0.381	0.417	0.436	0.399	0.376	0.386	0.409	0.398
MAE	0.327	0.353	0.294	0.292	0.290^e^	0.291 ^e^	0.316	0.331	0.361	0.302	0.279 ^e^	0.301	0.317	0.310
MedAE	0.278	0.28	0.234	0.231	0.221	0.228	0.215	0.248	0.292	0.194	0.185	0.236	0.26	0.257
Percentage of Eyes within Diopter Range Indicated														
± 0.25D	48.48	45.45	54.55	54.55	54.55	56.06	57.58	51.52	43.94	56.06	60.61	53.03	50	51.52
±0.50D	77.27	77.27	83.33	83.33	84.85	78.79	81.82	78.79	72.73	80.30	83.33	84.85	78.79	83.33
± 0.75D	86.36	86.36	96.97	95.45	93.94	92.42	90.91	92.42	89.39	90.91	90.91	93.94	92.42	92.42
± 1.00D	98.48	95.45	98.48	100.00	98.48	98.48	98.48	95.45	98.48	100.00	100.00	98.48	98.48	98.48

a, Heteroscedastic test, significantly lower than VRF, *P* < 0.05

b, Heteroscedastic test, significantly lower than BUII, *P* < 0.015

c, Heteroscedastic test, significantly lower than Olsen (OLCR), *P* < 0.015

d, Heteroscedastic test, significantly lower than Naeser, *P* < 0.025

e, Heteroscedastic test, significantly lower than Castrop, *P* < 0.025

In the group with corneal power less than 43 D (*n* = 65), VRF-G, EVO2.0, Kane, and K6 showed SDs of ± 0.370D, ± 0.379D, ± 0.380D, and ± 0.383D, respectively, and MAEs of 0.294D, 0.291D, 0.299D, and 0.294D. The proportion of eyes achieving a PE within ± 0.50D ranged from 81.54 to 86.15%. The BUII formula had an SD of ± 0.469D, higher compared to EVO2.0 and K6 (all *P* < 0.001). The VRF formula had higher SD of ± 0.468D than EVO2.0, Kane, Hoffer QST and VRF-G formulas (all *P* < 0.05).

In the subgroup with corneal power between 43 D and 46 D (*n* = 205), the RBF3.0, Karmona, K6, BUII, VRF-G, Pearl-DGS, Kane, and EVO2.0 formulas had SDs ranging from ± 0.405D to ± 0.417D and MAEs from 0.307D to 0.320D. The percentage of eyes within ± 0.50D PE for VRF-G and RBF3.0 was above 80.0%. Olsen (OLCR) and Castrop showed higher SDs of ± 0.470D and ± 0.450D, and MAEs of 0.368D and 0.356D, respectively. A statistical analysis of the SDs and MAEs revealed significant differences among all formulas in Table 3.

In the group with corneal power of 46 D or higher (*n* = 66), EVO2.0, RBF3.0, Hoffer QST, K6, and Kane had SDs between ± 0.375D and ± 0.384D. The RBF3.0, K6 and Kane also achieved the lowest MAEs (0.279D, 0.290D, 0.291D, respectively) and which were significantly lower than the Castrop (all *P* < 0.001). Olsen (OLCR) and Castrop recorded higher SDs of ± 0.436D and ± 0.448D, and higher MAEs of 0.361D and 0.353D, with the lowest percentages of eyes achieving PE within ± 0.25D and ± 0.50D.

## Discussion

This study conducted a comprehensive assessment of 14 newer IOL calculation formulas, with specific emphasis on their accuracy in predicting outcomes for different corneal curvatures. Our findings provide valuable insights into the nuanced performance of these formulas in relation to corneal power, offering valuable guidance for their appropriate application in specific corneal profiles.

Our analysis revealed that the VRF-G, EVO2.0, Kane, and K6 formulas demonstrate exceptional accuracy in eyes with flat corneal power. These formulas seem to effectively compensate for the unique optical characteristics presented by flat corneas. The VRF-G formula, which incorporates elements of theoretical optics, regression analysis, and ray tracing [13], contributing to its high precision in this group. This aligns with the accuracy levels reported in earlier studies [16, 17, 32]. Additionally, the K6 formula, which has not been as extensively studied as others, exhibited notable performance in eyes with short and long ALs, displaying results comparable to those of the Kane and EVO2.0 formulas [10, 17, 18]. Kane and EVO2.0 also performed well and have good stability in flat cornea group. This finding was consistent with many previous studies [16, 19, 33, 34]. 

The middle range of corneal curvature presented a different challenge, with formulas such as RBF3.0, Karmona, K6, BUII, VRF-G, Pearl-DGS, Kane, and EVO2.0 showing commendable accuracy. Interestingly, formulas like Pearl-DGS [10], despite being not better than the other new formulas in several studies [17, 34, 35], showcased promising results, comparable to established formulas like Kane and EVO2.0 in the medium K group. The Karmona formula was designed and programmed in Shiny-RStudio version 1.1.423 (R Foundation, Boston, USA) by David Carmona González [6] and reported better results (SD = ± 0.30D) than ours (SD = ± 0.415D). This variance could be attributed to our inability to obtain the mean keratometry of posterior surface to substitute into the calculations. To the best of our knowledge, few studies investigated the accuracy of Karmona formula in different range of corneal power and in our results, the Karmona formula showed good accuracy in the medium K group.

For steep corneal powers, our findings suggested a superior performance from formulas like RBF3.0, K6, and Kane. The EVO2.0 and Hoffer QST also performed well. The RBF3.0 formula was found to have good results at different corneal curvatures in our previous studies in long eyes [33]. The K6 formula showed its effectiveness in both flat and steep corneas, although its efficacy in long eyes with abnormal corneal power was somewhat less pronounced [33]. The Kane formula maintained its accuracy across eyes with abnormal corneal power, in both normal and long ALs, as supported by the results of this and previous studies [33]. 

Some limitations have been identified in this study. Firstly, we refrained from comparing classic formulas due to prior studies [1, 23] evaluating the efficacy of conventional formulas across various ranges of corneal curvature. Furthermore, the exclusion of more extreme ocular parameters, particularly in eyes with atypical ALs, might limit the applicability of our findings to a broader patient population. Also, additional ocular biology measurements should be obtained, such as the mean keratometry of posterior surface and total keratometry, which may have had a impact on the postoperative refraction and needs more investigation.

In summary, our study offers valuable insights into the performance of various newer-generation IOL calculation formulas across different corneal curvature groups. The VRF-G, EVO2.0, Kane, K6 showed good accuracy in flat K eyes, while the RBF3.0, K6 and Kane performed better in eyes with steep corneal power.

## Data Availability

The datasets generated during and/or analyzed during the current study are available from the corresponding author on reasonable request.
